# Analysis of *DNM3* and *VAMP4* as genetic modifiers of *LRRK2* Parkinson’s disease

**DOI:** 10.1016/j.neurobiolaging.2020.07.002

**Published:** 2021-01

**Authors:** Emmeline E. Brown, Cornelis Blauwendraat, Joanne Trinh, Mie Rizig, Mike A. Nalls, Etienne Leveille, Jennifer A. Ruskey, Hallgeir Jonvik, Manuela M.X. Tan, Sara Bandres-Ciga, Sharon Hassin-Baer, Kathrin Brockmann, Jon Infante, Eduardo Tolosa, Mario Ezquerra, Sawssan Ben Romdhan, Mustapha Benmahdjoub, Mohamed Arezki, Chokri Mhiri, John Hardy, Andrew B. Singleton, Roy N. Alcalay, Thomas Gasser, Donald G. Grosset, Nigel M. Williams, Alan Pittman, Ziv Gan-Or, Ruben Fernandez-Santiago, Alexis Brice, Suzanne Lesage, Matthew Farrer, Nicholas Wood, Huw R. Morris, International Parkinson Disease Genomics Consortium (IPDGC)

**Affiliations:** a Department of Clinical and Movement Neurosciences, Institute of Neurology, University College London, London, UK; b Laboratory of Neurogenetics, National Institute on Aging, National Institutes of Health, Bethesda, MD, USA; c Institute of Neurogenetics, University of Lübeck, Lübeck, Germany; d Data Tecnica International, Glen Echo, MD, USA; e Montreal Neurological Institute, McGill University, Montréal, Quebec, Canada; f Department of Neurology and Neurosurgery, McGill University, Montréal, Quebec, Canada; g Instituto de Investigación Biosanitaria de Granada (ibs.GRANADA), Granada, Spain; h Sackler Faculty of Medicine, Tel Aviv University, Tel Aviv, Israel; i Department of Neurology, Sheba Medical Center, Tel HaShomer, Israel; j Movement Disorders Institute, Sheba Medical Center, Tel HaShomer, Israel; k Hertie Institute for Clinical Brain Research and German Center for Neurodegenerative Diseases, University Clinic Tu¨bingen, Tu¨bingen, Germany; l Service of Neurology, University Hospital “Marqués de Valdecilla (IDIVAL)”, University of Cantabria; m “Centro de Investigación Biomédica en Red de Enfermedades, Neurodegenerativas (CIBERNED)”, Santander, Spain; n Laboratory of Parkinson Disease & Other Neurodegenerative Movement Disorders, Institut d'Investigacions Biomèdiques August Pi i Sunyer (IDIBAPS) - Hospital Clínic de Barcelona, Barcelona, Spain; o Centro de Investigación Biomédica en Red sobre Enfermedades Neurodegenerativas (CIBERNED: CB06/05/0018-ISCIII), Barcelona, Spain; p Research Unit in Neurogenetics, Clinical Investigation Center (CIC) at the CHU Habib Bourguiba, Sfax, Tunisia; q Frantz Fanon Hospital, CHU Blida, Blida, Algeria; r Department of Neurodegenerative Disease, UCL Queen Square Institute of Neurology, Queen Square, London WC1N 3BG, UK; s UK Dementia Research Institute at UCL and Department of Neurodegenerative Disease, UCL Institute of Neurology, University College London, London, UK; t Reta Lila Weston Institute, UCL Queen Square Institute of Neurology, 1 Wakefield Street, London WC1N 1PJ, UK; u UCL Movement Disorders Centre, University College London, London, UK; v Institute for Advanced Study, The Hong Kong University of Science and Technology, Hong Kong SAR, China; w Department of Neurology, College of Physicians and Surgeons, Columbia University, New York, NY, USA; x Taub Institute for Research on Alzheimer’s Disease and the Aging Brain, Columbia University, New York, NY, USA; y Department for Neurodegenerative Diseases, Hertie Institute for Clinical Brain Research, University of Tu¨bingen, Tu¨bingen, Germany; z German Center for Neurodegenerative Diseases (DZNE), Tu¨bingen, Germany; aa Department of Neurology, Institute of Neurological Sciences, Queen Elizabeth University Hospital, Glasgow, UK; bb Institute of Neuroscience & Psychology, University of Glasgow, Glasgow, UK; cc Division of Psychological Medicine & Clinical Neuroscience, School of Medicine, Cardiff University, Cardiff, UK; dd Department of Clinical Genetics, St George’s, University of London, London, UK; ee Department of Human Genetics, McGill University, Montreal, Quebec, Canada; ff Research Unit U1127 at INSERM, Research Unit UMR 7225 at the French National Centre for Scientific Research (CNRS) Research Unit UMR_1127 at Sorbonne Université, Institutet du Cerveau et de la Moëlle épinière (ICM), Paris, France; gg Department of Medical Genetics, University of British Columbia, Vancouver, British Columbia, Canada

**Keywords:** Parkinson’s disease, Genetic modifiers, Parkinsonism, Leucine-rich repeat kinase 2

## Abstract

The *LRRK2* gene has rare (p.G2019S) and common risk variants for Parkinson’s disease (PD). *DNM3* has previously been reported as a genetic modifier of the age at onset in PD patients carrying the *LRRK2* p.G2019S mutation. We analyzed this effect in a new cohort of *LRRK2* p.G2019S heterozygotes (*n* = 724) and meta-analyzed our data with previously published data (*n* = 754). *VAMP4* is in close proximity to *DNM3*, and was associated with PD in a recent study, so it is possible that variants in this gene may be important. We also analyzed the effect of *VAMP4* rs11578699 on *LRRK2* penetrance. Our analysis of *DNM3* in previously unpublished data does not show an effect on age at onset in *LRRK2* p.G2019S carriers; however, the inter-study heterogeneity may indicate ethnic or population-specific effects of *DNM3*. There was no evidence for linkage disequilibrium between *DNM3* and *VAMP4*. Analysis of sporadic patients stratified by the risk variant *LRRK2* rs10878226 indicates a possible interaction between common variation in *LRRK2* and *VAMP4* in disease risk.

## Highlights

• Analysis of *DNM3* in previously unpublished data does not show an interaction between *DNM3* and *LRRK2* G2019S for AAO.
• There was no evidence for linkage disequilibrium between *DNM3* rs2421947 and *VAMP4* rs11578699.
• Analysis of sporadic PD patients stratified by the PD risk variant rs10878226 indicates a possible interaction between *LRRK2* and *VAMP4.*

## 1 Introduction

The p.G2019S coding variant in the *LRRK2* (leucine-rich repeat kinase 2) gene is the commonest high penetrance mutation causing parkinsonism. The mutation occurs in 1%–40% of Parkinson’s disease (PD) cases, varying by ethnicity (Healy et al., 2008). *LRRK2* parkinsonism is broadly similar to “idiopathic” disease in clinical manifestations and age at onset (AAO), generating interest in its potential as a therapeutic target with broader application to PD (Di Maio et al., 2018). Genome-wide association studies (GWAS) show that common variation in *LRRK2* is also a risk factor for sporadic PD (Mata et al., 2012; Ross et al., 2011).

There is a wide range in PD AAO among p.G2019S carriers. It has been reported that the PD AAO of *LRRK2* p.G2019S carriers is modified by the Dynamin-3 (*DNM3*) rs2421947 variant; GG rs2421947 homozygotes have been reported to have a median disease onset 12.5 years younger than other p.G2019S carriers (Trinh et al., 2016). *DNM3* is a microtubule-associated protein that is able to bind and hydrolyze guanosine triphosphate and is involved in producing microtubule bundles. The mechanism through which it may impact PD AAO for *LRRK2* patients is not understood. In a cohort from Spain the median onset was 3 years younger in patients with the G allele, though this was not significant (Fernandez-Santiago et al., 2018). There was no effect of *DNM3* on PD AAO in individuals carrying Asian *LRRK2* risk alleles (Foo et al., 2019).

In the largest GWAS meta-analysis of idiopathic PD (iPD) the nearby *VAMP4* (vesicle-associated membrane protein 4) rs11578699, located 113,325 bp from *DNM3* rs2421947, is associated with PD, raising the possibility that the effect of *DNM3* may relate to linkage disequilibrium with *VAMP4* (Nalls et al., 2019). It has been suggested that *VAMP4* may be involved in PD through the endocytic membrane trafficking pathway.

We sought to replicate the *DNM3* discovery finding, to determine whether the association varies with ethnicity and whether linkage disequilibrium with *VAMP4* is relevant. We analyzed a multi-ethnic cohort of *LRRK2* p.G2019S carriers and a larger cohort of European patients with and without the *LRRK2* common risk allele.

## 2 Methods

### 2.1 Data collection

#### 2.1.1 Patient cohorts

*LRRK2* p.G2019S heterozygote participants were identified from cohorts in the International Parkinson’s Disease Genomics Consortium (IPDGC) and other collaborative centers. Data from 724 *LRRK2* p.G2019S heterozygote participants were contributed by the National Institute of Health, University College London, McGill University (samples were collected in Columbia University and Sheba Medical Center), Sorbonne University (in collaboration with Habib Bourguiba Hospital, Sfax, Tunisia, and Blida Hospital, Blida, Algeria), National Institute of Neurological Disorders and Stroke and the University of Tübingen. Data from 2 published studies analyzing *DNM3* rs2421947 and *LRRK2* p.G2019S parkinsonism with AAO was also included comprising an additional 754 participants (Fernandez-Santiago et al., 2018; Trinh et al., 2016). All studies willing to participate and currently holding minimum required data for *LRRK2* p.G2019S carriers were included: PD onset age or sampling age for asymptomatic carriers; and *DNM3* rs2421947 or a high r^2^ (measure of linkage disequilibrium [LD]) proxy single nucleotide polymorphism. Where these studies had also genotyped *VAMP4* rs11578699 or a high r^2^ proxy, these data were also included in the analysis of the *VAMP4* gene (*n* = 786). Patients with PD without a known Mendelian or high-risk genetic cause of disease were collated from the UK-wide *Tracking Parkinson’s* study (Malek et al., 2015) and from IPDGC datasets. Summary statistics for *DNM3* rs2421947 and *VAMP4* rs11578699 were obtained from the most recent large-scale PD GWAS (Nalls et al., 2019) and PD AAO GWAS (Blauwendraat et al., 2019).

#### 2.1.2 Standard protocol approvals, registrations, and patient consents

All studies were approved by each respective Institutional Ethics Review Committee and all participants provided written informed consent. All studies were carried out in accordance with the Declaration of Helsinki.

Research ethics approval for the *Tracking Parkinson’s study* was provided by West of Scotland Research Ethics Service (reference 11/AL/0163). The study is registered with ClinicalTrials.gov under the identifier NCT02881099. Research ethics approval for the *Parkinson’s Families Project* was provided by the London Camden and King’s Cross ethics committee (reference 15/LO/0097) and the Health Research Authority. The study is registered with ClinicalTrials.gov under the identifier NCT02760108. The local Research Ethics Board number for samples processed at McGill University is IRB00010120, and the approved project identifier is 2017-2944, valid until December 2019, to be renewed then. The local Institutional Review Board approval for samples from Columbia University is AAAF3108. Ethical approval from 2 of the studies is published (Fernandez-Santiago et al., 2018; Trinh et al., 2016).

### 2.2 Genetic analyses

Genotyping was performed on different platforms (NeuroChip array, NeuroX array, TaqMan assays, Infinium OmniExpress-24, and Illumina HumanCore Exome array) at participating centers. The *LRRK2* p.G2019S mutation was directly genotyped. Sanger sequence or KASPar (“Kompetitive” allele-specific polymerase chain reaction) verification of the *DNM3* rs2421947 variant was carried out on data subsets at study-specific centers. Where the variant *DNM3* rs2421947 was imputed, only r^2^ values above 0.85 were used, and only r^2^ values above 0.75 were used for *VAMP4* rs11578699 (Table e1), as the r^2^ quality of available data for variant *VAMP4* rs11578699 was lower. In total, 1478 *LRRK2* p.G2019S carriers were identified across study cohorts. In addition, 2052 samples from patients with iPD from the Tracking Parkinson’s disease study passed quality control and had no identified genetic cause of disease (Malek et al., 2015). Ninety-six patients with PD were excluded due to missing AAO data; therefore 1956 patients with PD and without *LRRK2* p.G2019S were included in further analysis. Summary statistics and patient data from the most recent PD GWAS included over 37.7K cases, 18.6K “proxy-cases,” and 1.4M controls. The most recent PD AAO GWAS included data from 28.6K patients (Blauwendraat et al., 2019; Nalls et al., 2019).

### 2.3 Statistical analyses

**Fig. 1:**
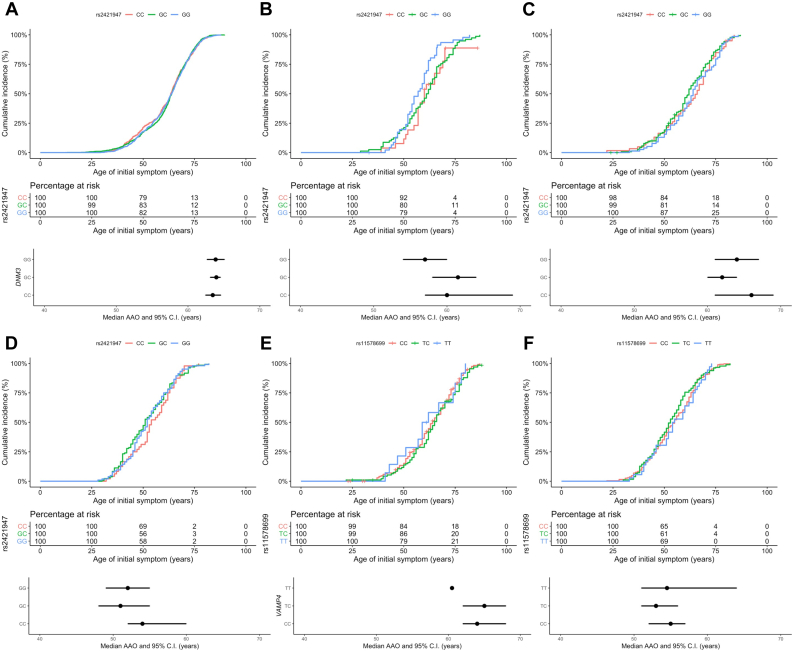
Survival analysis of AAO in *DNM3* rs2421947 (A–D) and *VAMP4* rs11578699 genotype groups (E, F). *DNM3* rs2421947 Kaplan-Meier and Kaplan-Meier median plots of (A) European idiopathic PD (n = 1956); (B) Ashkenazi Jewish p.G2019S carriers (n = 153) Kaplan-Meier plot and median plot; (C) European p.G2019S carriers (n = 286); and (D) North African p.G2019S carriers (n = 285). *VAMP4* rs2421947 Kaplan-Meier and Kaplan-Meier median plots of (E) European and Ashkenazi Jewish p.G2019S carriers (n = 302); and (F) North African p.G2019S carriers (n = 484). Abbreviations: AAO, age at onset; *DNM3*, Dynamin 3; PD, Parkinson’s disease; *VAMP4*, vesicle-associated membrane protein 4.

**Fig. 2:**
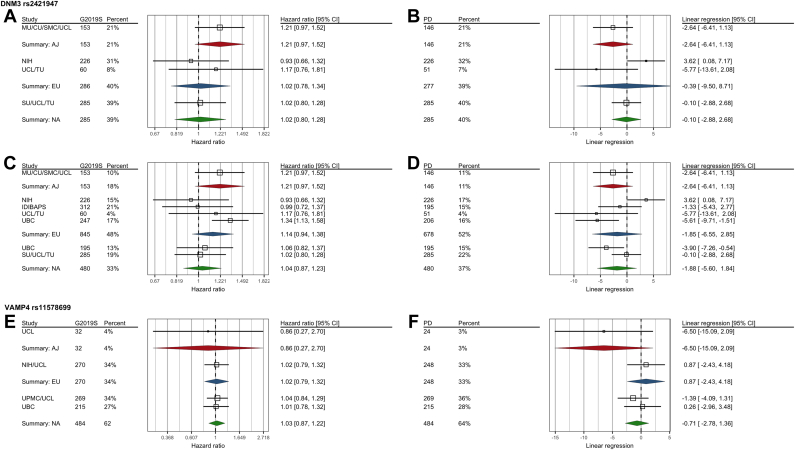
Forest plot of meta-analyses for *DNM3* rs2421947 and *VAMP4* rs11578699. (A, C, and E) Cox proportional hazards model meta-analyses of *LRRK*2 p.G2019S carriers; (B, D, and F) linear regression meta-analyses of *LRRK*2 p.G2019S patients. (A, B) Meta-analyses of *DNM3* rs2421947 GG versus CG and CC genotypes of novel data from this manuscript from 724 *LRRK2* p.G2019S carriers; (C, D) Meta-analyses of *DNM3* rs2421947 GG versus CG and CC genotypes from 1478 *LRRK2* p.G2019S carriers including 754 previously published; (E, F) *VAMP4* rs11578699 meta-analyses of CC versus TT and TC. Percentage contribution and numbers of individuals included in each analysis are indicated. Analyses are carried out on ethnicity subgroups: Ashkenazi Jewish (Summary: AJ), European (Summary: EU), and North African (Summary: NA). Abbreviations: *DNM3*, Dynamin 3; *LRRK2*, leucine-rich repeat kinase 2; *VAMP4*, vesicle-associated membrane protein 4.

Data from the studies were pooled and principal component analysis was used to define ethnicity, where available. Within the Ashkenazi Jewish (AJ), European, and North African analyses ethnic outliers were excluded. We calculated Hardy-Weinberg equilibrium for *VAMP4* rs11578699 and *DNM3* rs2421947 in p.G2019S carriers. Quality control Hardy-Weinberg equilibrium for iPD and controls is summarized in the recent large-scale PD GWAS. We assessed linkage disequilibrium (LD) measures r^2^ and D′ between *DNM3* rs2421947 and *VAMP4* rs11578699 in different populations to evaluate the possibility that an extended haplotype block might explain the relationship between *DNM3* and *LRRK2* (Table e2). The allele-based Fisher’s exact test was used to compare *DNM3* rs2421947 and *VAMP4* rs11578699 minor allele frequencies between *LRRK2* p.G2019S heterozygotes of different ethnic backgrounds and with iPD (Table 1). Student’s *t*-tests and analysis of variance were used to assess differences in AAO by genotype (Table 2). We then analyzed the effect of *DNM3* rs2421947 (Fig. 1) on AAO in iPD in the *Tracking Parkinson’s* dataset using linear regression of AAO, and Kaplan-Meier survival analysis. Next, we analyzed patients with *LRRK2* p.G2019S of AJ, North African, and European ethnicity using linear regression of AAO with available covariates of sex, ethnicity, relatedness, and study center of origin (Fig. 2). AAO was regressed by *DNM3* rs2421947 genotypes, and separately for rs11578699 *VAMP4* genotypes. We meta-analyzed *DNM3* rs2421947 data from all previously unpublished datasets using linear regression models (Fig. 2B). We pooled these data with previously published data and meta-analyzed again with the same methods (Fig. 2C and D). We then analyzed *VAMP4* rs11578699 effect on AAO with linear regression (Fig. 2E and F). Bonferroni or other corrections for multiple testing were not performed as this is a candidate gene-based study. Finally, we analyzed the effect of *VAMP4* rs11578699 on AAO in iPD carrying the *LRRK2* rs10878226 variant. *LRRK2* rs10878226 has also been implicated in PD risk (odds ratio 1.20, 95% confidence interval [CI] 1.08–1.33, *p* = 6.3 × 10^−4^, *n* = 6129) (Mata et al., 2012).

**Table 1:** Minor allele frequency for *DNM3* rs2421947 and *VAMP4* rs11578699 in PD cases and controls with and without p.G2019S

	gnomAD^a^	p.G2019S PD^b^	p.G2019S asymptomatic^c^	Idiopathic PD	Total^f^
*DNM3* rs2421947					
Ashkenazi Jewish	0.47 (*n* = 145)	0.41 (*n* = 146)	0.79 (*n* = 7)	N/A	0.45 (*n* = 298)
African/North African	0.42 (*n* = 4349)	0.39 (*n* = 480)	N/A	N/A	0.42 (*n* = 4829)
From Spain/Latino	0.41 (*n* = 424)	0.49 (*n* = 195)	0.49 (*n* = 117)	N/A	0.44 (*n* = 736)
European (non-Finnish)	0.41 (*n* = 7700)^d^	0.47 (*n* = 483)	0.68 (*n* = 50)	0.44 (*n* = 14,508)	0.43 (*n* = 22,734)
Total	0.41 (*n* = 12,618)	0.44 (*n* = 1304)	0.56 (*n* = 174)	0.44 (*n* = 14,508)	N/A
*VAMP4* rs11578699					
Ashkenazi Jewish	0.20 (*n* = 138)	0.27 (*n* = 24)	0.5 (*n* = 8)	N/A	0.22 (*n* = 170)
African/North African	0.18 (*n* = 3979)	0.26 (*n* = 484)^e^	N/A	N/A	0.19 (*n* = 4463)
From Spain/Latino	0.16 (*n* = 393)	N/A	N/A	N/A	0.16 (*n* = 393)
European (non-Finnish)	0.19 (*n* = 6994)	0.20 (*n* = 248)	0.18 (*n* = 22)	0.19 (*n* = 21,242)	N/A
Total	0.18 (*n* = 11,504)	0.23 (*n* = 752)	0.27 (*n* = 30)	0.19 (*n* = 21,242)	N/A

Key: *DNM3*, Dynamin 3; *LRRK2*, leucine-rich repeat kinase 2; N/A, not available; PD, Parkinson’s disease; *VAMP4*, vesicle-associated membrane protein 4.

a The gnomAD database features unrelated individuals sequenced as part of disease-specific and population-based genetic studies. Individuals affected by severe pediatric disease and their first-degree relatives have not been included.

b *LRRK2* p.G2019S parkinsonism.

c *LRRK2* p.G2019S asymptomatic carriers.

d This series includes ethnicity subcategories: Estonian, other non-Finnish European, Northwestern European, and Southern European.

e North African *LRRK*2 p.G2019S parkinsonism.

f All samples from the following categories were combined: gnomAD, idiopathic PD, *LRRK2* p.G2019S patients, and asymptomatic carriers.

**Table 2:** Age at onset by genotype in *LRRK2* p.G2019S carriers in this study

*DNM3* rs2421947							
Population series	Mean age at onset (SD)			ANOVA *p* value	GG vs. CG *p* value^a^	CG vs. CC *p* value^a^	GG vs. CC *p* value^a^
	GG	CG	CC				
Ashkenazi Jewish (*n* = 146)	57.20 (8.96)	60.11 (12.21)	58.41 (8.01)	0.34	0.13	0.45	0.58
North African (*n* = 285)	52.64 (11.21)	51.95 (11.99)	54.27 (11.05)	0.49	0.65	0.23	0.40
European^b^ (*n* = 277)	63.26 (12.14)	61.20 (12.45)	62.17 (13.48)	0.52	0.24	0.64	0.63
Combine(d^c^*n* = 708)	57.11 (12.02)	57.44 (12.93)	58.59 (12.30)	0.55	0.76	0.37	0.28
*VAMP4* rs11578699							
Population series	CC	TC	TT	ANOVA *p* value	CC vs. TC *p* value^a^	TC vs. TT *p* value^a^	CC vs. TT *p* value^a^
Ashkenazi Jewish (*n* = 24)	63.92 (9.57)	57.45 (11.93)	57.00 (N/A)	0.91	0.17	N/A	N/A
North African (*n* = 484)	54.38 (11.89)	53.35 (11.58)	55.22 (11.48)	0.55	0.36	0.38	0.68
European^b^ (*n* = 248)	62.44 (12.38)	63.89 (12.21)	61.33 (13.75)	0.66	0.41	0.55	0.79
Combined^c^ (*n* = 756)	57.39 (12.61)	56.41 (12.62)	56.76 (12.10)	0.60	0.32	0.85	0.73

Combined subgroup mean and standard deviation age at onset in years of *LRRK2* p.G2019S parkinsonism for *DNM3* rs2421947 and *VAMP4* rs11578699 genotypes; one-way ANOVA between *DNM3* rs2421947 and *VAMP4* rs11578699 genotypes; Student’s *t*-test between genotype.

Key: ANOVA, analysis of variance; *DNM3*, Dynamin 3; *LRRK2*, leucine-rich repeat kinase 2; N/A, not available; *VAMP4*, vesicle-associated membrane protein 4.

a Uncorrect.

b The following population series subgroups were from combined European unspecific and British.

c The following population series subgroups were from combined Ashkenazi Jewish, North African, European unspecific, and British.

### 2.4 Gene expression

Gene expression profiles of *DNM3* rs2421947 and *VAMP4* rs11578699 were assessed using publicly available Web-based resources: BRAINEACv.2 (www.braineac.org) (Ramasamy et al., 2014); GTEx (www.gtexportal.org); and Allen Brain Atlas (www.brain-map.org) (Hawrylycz et al., 2012), and evaluated based on the significance level required for inclusion in colocalization (COLOC) analysis (Appendix e-1).

### 2.5 Data availability statement

The majority of anonymized data not published in full here will be shared by request from any qualified investigator, with the exception of a subset of data from the latest IPDGC GWAS which cannot be shared for legal or ethical reasons.

## 3 Results

*DNM3* did not influence AAO in all PD patients from the Tracking Parkinson’s cohort (GG vs. CC and CG carriers: beta = −0.02, *p* = 0.97, *n* =1956). This is consistent with the latest PD AAO GWAS (*p* = 0.39, effect = −0.11, standard error [se] = 0.13). The Kaplan-Meier method was used for visualizing risk across *DNM3* rs2421947 genotypes for iPD (Fig. 1A), and *LRRK2* p.G2019S carriers (Fig. 1B–D). The same method was also used to visualize risk for *VAMP4* rs11578699 genotypes in individuals with *LRRK2* p.G2019S (Fig. 1E and F).

We carried out a multi-ethnic meta-analysis of newly contributed data of *DNM3* rs2421947 GG versus CC and CG genotypes against onset age in 724 *LRRK2* p.G2019S carriers, using a random effects model on disease-free survival. There was no effect of *DNM3* on age at onset in G2019S carriers (linear regression was not significant, beta = −1.19, *p* = 0.55, *n* = 708), though there was considerable heterogeneity between studies (*I*^2^ = 86.9%, *p* < 0.01). The effect of GG versus CC and CG was not significant in sub-group analyses (Fig. 2).

When our new data were pooled with the 2 previous studies and meta-analyzed, linear regression meta-analysis of *LRRK2* p.G2019S AAO was not significant (beta = −2.21, *p* = 0.083, *n* = 1304). There was heterogeneity between studies (*I*^2^ = 82.3%, *p* < 0.0001) (Fig. 2).

Meta-analysis of the effect of *VAMP4* rs11578699 (the genome-wide significant association in a recent large-scale PD GWAS) on AAO in *LRRK*2 p.G2019S parkinsonism was not significant for CC versus TT and TC genotypes (beta = −0.25, *p* = 0.75, *n* = 756).). *I*^2^ total heterogeneity was <0.001%, *p* = 0.75 (Fig. 2).

We evaluated the possibility of ethnic-specific effects using linear regression of AAO (Fig. 2). Though in each subgroup analysis confidence intervals overlapped zero, there appeared to be a trend toward earlier onset in G allele carriers of AJ ethnicity, consistent with previously reported data, and in the discovery data effects were strongest in Arab-Berbers suggesting ethnic-specific effects.

Using Cox proportional survival analysis, we studied the discovery data and subsequent cohorts separately (GG vs. CC and CG as described previously). There was a strong association between rs2421947 and PD AAO in discovery data (hazard ratio [HR] 1.59, 95% CI 1.28–1.97, *p* < 0.001). This was also present through AAO linear regression analysis (beta = −4.93, *p* = 0.00019). The association was absent in our replication data (HR 1.09, 95% CI 0.95–1.26, *p* = 0.22). We meta-analyzed the samples studied in this paper with the discovery data (Fig. 2).

**Fig. 3:**
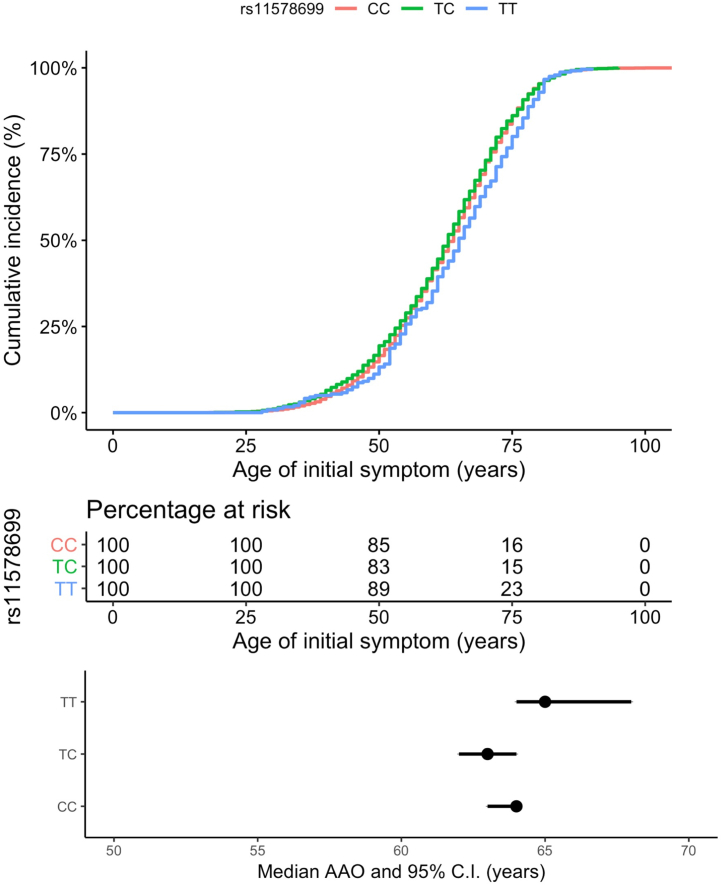
Kaplan-Meier analysis by *VAMP4* rs11578699 genotype in PD cases carrying *LRRK2* risk variant (rs10878226). Abbreviations: *LRRK2*, leucine-rich repeat kinase 2; PD, Parkinson’s disease; *VAMP4*, vesicle-associated membrane protein 4.

Finally, we investigated the possibility that there might be an interaction between the GWAS defined common *LRRK2* risk allele (rs10878226) and *VAMP4*. We analyzed 4882 cases with PD carrying the *LRRK2* risk variant minor allele using linear regression of AAO, and plotting of the Kaplan-Meier curve, as shown in Fig. 3. TT versus TC and CC *VAMP4* rs11578699 genotype was nominally significantly associated with AAO risk in this cohort (beta = 1.68, se = 0.81, *p* = 0.037, *n* = 4882*). VAMP4* rs11578699 was not significantly associated with AAO risk in iPD cases without the *LRRK2* rs10878226 risk variant (beta = −0.28, se = 0.48, *p* = 0.56, *n* = 14,970). Far fewer patients with iPD had been genotyped for *DNM3* rs2421947 in the PD GWAS cohort (Table 1), though for *DNM3* rs2421947 was not associated with AAO risk in iPD cases with the *LRRK2* rs10878226 risk variant (beta = 0.045, se = 0.53, *p* = 0.93, *n* = 3300) nor without (beta = 0.21, se = 0.31, *p* = 0.69, *n* = 9822).

## 4 Discussion

We have analyzed the effect of *DNM3* rs2421947 on AAO in *LRRK2* p.G2019S parkinsonism.

We have not replicated the association between *DNM3* and *LRRK2* p.G2019S AAO in this study or in meta-analysis with all available data. In the original discovery analysis, there was a difference of 12.5 years between *DNM3* rs2421947 GG and CC genotypes (meta-analysis HR 1.61, 95% CI 1.15–2.27, *p* = 0.02). In our data the AAO difference between GG and CC genotypes was 1.4 years, which was not significant. Using AAO regression, meta-analysis of independent sample series in newly genotyped samples did not identify a significant difference in AAO between genotypes (beta = −1.19, *p* = 0.55, *n* =708). Similarly, when discovery and replication data were analyzed together there was no significant effect of *DNM3* rs2421947 on AAO (beta = −2.21, *p* = 0.083, *n* = 1304). However, there was significant heterogeneity in replication and combined *DNM3* regression meta-analyses.

In “sporadic” PD, consistent with the recent AAO GWAS (Ross et al., 2011), our study indicated that *DNM3* rs2421947 does not affect AAO in non-p.G2019S disease.

We did not identify LD between DNM3 and *VAMP4*, nor an independent effect of *VAMP4* on *LRRK2* penetrance. Analysis of carriers of a common *LRRK2* risk single nucleotide polymorphism provided nominally significant support for a potential interaction between *VAMP4* and *LRRK2*, which requires replication in larger sample sizes. Gene expression analysis indicated that *VAMP4* rs11578699 is an expression quantitative trait loci for *VAMP4* expression.

One possibility for the lack of replication of *DNM3* modification of p.G2019S AAO is that heterogeneity in the sample may limit the observed effect in the meta-analysis; this has previously led to non-replication in independent samples in GWAS (Dunckley et al., 2007; Fernandez-Santiago et al., 2011). Significant heterogeneity was observed in the linear regression meta-analysis of replication and combined data in this study, which may be explained by ethnicity effects. The effect of *DNM3* may vary between ethnicities or be population specific, and the strongest effects were seen in AJ p.G2019S carriers although not reaching significance (Fig. 2). Further studies in larger numbers of AJ and Arab patients are needed.

Due to its role in innate immunity *LRRK2* may have been under different selective pressures in human evolution, relating to differences in environmental pathogens. Interestingly, *LRRK2* p.G2019S penetrance varies across ethnic groups. Hentati et al. (2014) have reported a later AAO in *LRRK2* p.G2019S carriers from Norway compared to Tunisian patients. Later AAO penetrance for AJ PD p.G2019S carriers compared to non-Jewish carriers has been indicated previously through plotting of estimated cumulative risk, though this was not significant; penetrance was 42.5% (95% CI 26.3–65.8) in non-AJ relatives compared to 25% (95% CI 16.7–34.2) in AJ heterozygous relatives (Lee et al., 2017). These data imply that there may be potential protective factors in the relatively homogeneous Norwegian and AJ population and that ethnic and population effects are likely to be very important in analyzing this variant. Our analysis and comparison of background allele frequencies in this study suggest that this is unlikely to primarily relate to *VAMP4* and *DNM*3. However, there may be other rare variant effects that will emerge with fine mapping of this region.

The *LRRK2* common risk variant analysis is of interest; the variant is not a proxy for p.G2019S in Europeans, so this represents an independent marker of PD risk. Around a quarter (24.6%) of sporadic PD cases carry the common *LRRK2* risk variant rs10878226, which is associated with PD (combined odds ratio 1.20, 95% CI 1.08–1.33, *p* = 6.3 × 10^−4^, *n* = 6129) (Mata et al., 2012). Our common *LRRK2* variant analysis indicates a possible interaction between common variation in *LRRK2* and *VAMP4*, which requires further study. *VAMP4* and *LRRK2* are both involved in synaptic vesicle dynamics, which has relevance to the etiology of PD. Underscoring this possibility, analysis of *VAMP4* and *DNM3* expression indicated that both are highly expressed in the brain, though these are not in LD and likely represent independent signals.

Modifiers of the penetrance of *LRRK2* p.G20129S are likely to be therapeutic targets and may be important in genetic counseling. Our large study aggregating new and previously published data has indicated significant heterogeneity across studies. We have not replicated the original discovery of an interaction between *DNM3* and *LRRK2* p.G2019S. Further genome-wide studies in different populations are needed, to resolve the determinants of the variable penetrance seen in individuals with p.G2019S.

## Disclosure statement

Nalls’ participation in this project is part of a consulting contract between the National Institute on Aging and Data Tecnica International, LLC. He also consults for Lysosomal Therapeutics Inc, Neuron 23 Inc, Illumina Inc, the Michael J. Fox Foundation, and Aspen Neurosciences. Brockmann has received a research grant from the University of Tübingen (Clinician Scientist) and the German Society of Parkinson’s disease (dpv), funding from the Michael J. Fox Foundation (MJFF) and the German Centre for Neurodegenerative Diseases (DZNE, MIGAP), travel grants from the Movement Disorders Society, and speaker honoraria from AbbVie, Lundbeck, UCB, and Zambon. Dr Tolosa received honoraria for consultancy from Novartis, TEVA, Bial, Accorda, Boehringer Ingelheim, UCB, Solvay, Lundbeck, and BIOGEN and has received funding for research from Spanish Network for Research on Neurodegenerative Disorders (CIBERNED)-Instituto Carlos III (ISCIII), and The Michael J. Fox Foundation for Parkinson’s Research (MJFF). Dr Alcalay’s research is funded by the National Institute of Health, the Parkinson’s Foundation, and the Michael J. Fox Foundation. He received consultation fees from Genzyme/Sanofi, Restorbio, and Roche. Dr Grosset has received honoraria from Merz Pharma Vectura plc, and consultancy fees from The Glasgow Memory Clinic. Dr Gan-Or’s research is supported by grants from Parkinson Canada, the Michael J. Fox Foundation, the Canadian Consortium on Neurodegeneration in Aging (CCNA), the Canadian Glycomics Network (GlycoNet), the Canada First Research Excellence Fund (CFREF), awarded to McGill University for the Healthy Brains for Healthy Lives (HBHL) program, the Fonds de recherche du Québec - Santé (FRQS) Chercheurs-boursiers, and Parkinson Quebec. Dr Gan-Or received consultation fees from Denali, Inception Sciences, Idorsia, Lysosomal Therapeutics Inc, and Prevail Therapeutics. In the last 12 months, Dr Brice received grants from ENP-Ecole des Neurosciences Paris, Institut de France, ANR-EPIG and France Parkinson and FRC. Dr Farrer has received royalty payments from Athena Diagnostics for USA Patent 7,544,786 related to LRRK2 Gly2019Ser. Mayo Foundation and MJF have also received royalties from H Lundbeck and Merck related to the development of LRRK2 murine models including LRRK2 Gly2019Ser. Dr Morris is employed by University College London. In the last 12 months he reports paid consultancy from Biogen, UCB, AbbVie, Denali, and Biohaven; lecture fees/honoraria from Biogen, UCB, C4X Discovery, GE-Healthcare, Wellcome Trust, Movement Disorders Society; research grants from Parkinson’s UK, Cure Parkinson’s Trust, PSP Association, CBD Solutions, Drake Foundation, and Medical Research Council. Dr Morris is a co-applicant on a patent application related to C9ORF72—Method for diagnosing a neurodegenerative disease (PCT/GB2012/052140). The remaining authors disclose no conflicts.

## Appendix A Supplementary data

References (Chang et al. (2015), Kossmeier (2019), Seber and Wild (1989), Therneau (2015) and Viechtbauer (2010)) are cited in the Supplementary Methods.

Figure E2 1

Figure E3 2

Supplementary methods

Figure E1

## References

[bib1] Blauwendraat C., Heilbron K., Vallerga C.L., Bandres-Ciga S., von Coelln R., Pihlstrom L., Simon-Sanchez J., Schulte C., Sharma M., Krohn L., Siitonen A., Iwaki H., Leonard H., Noyce A.J., Tan M., Gibbs J.R., Hernandez D.G., Scholz S.W., Jankovic J., Shulman L.M., Lesage S., Corvol J.C., Brice A., van Hilten J.J., Marinus J., Eerola-Rautio J., Tienari P., Majamaa K., Toft M., Grosset D.G., Gasser T., Heutink P., Shulman J.M., Wood N., Hardy J., Morris H.R., Hinds D.A., Gratten J., Visscher P.M., Gan-Or Z., Nalls M.A., Singleton A.B., 23andMe Research, International Parkinson's Disease Genomics Consortium (2019). Parkinson’s disease age at onset genome-wide association study: defining heritability, genetic loci, and alpha-synuclein mechanisms. Mov. Disord..

[bib2] Chang C.C., Chow C.C., Tellier L.C., Vattikuti S., Purcell S.M., Lee J.J. (2015). Second-generation PLINK: rising to the challenge of larger and richer datasets. Gigascience.

[bib3] Di Maio R., Hoffman E.K., Rocha E.M., Keeney M.T., Sanders L.H., De Miranda B.R., Zharikov A., Van Laar A., Stepan A.F., Lanz T.A., Kofler J.K., Burton E.A., Alessi D.R., Hastings T.G., Greenamyre J.T. (2018). LRRK2 activation in idiopathic Parkinson’s disease. Sci. Transl. Med..

[bib4] Dunckley T., Huentelman M.J., Craig D.W., Pearson J.V., Szelinger S., Joshipura K., Halperin R.F., Stamper C., Jensen K.R., Letizia D., Hesterlee S.E., Pestronk A., Levine T., Bertorini T., Graves M.C., Mozaffar T., Jackson C.E., Bosch P., McVey A., Dick A., Barohn R., Lomen-Hoerth C., Rosenfeld J., O'Connor D.T., Zhang K., Crook R., Ryberg H., Hutton M., Katz J., Simpson E.P., Mitsumoto H., Bowser R., Miller R.G., Appel S.H., Stephan D.A. (2007). Whole-genome analysis of sporadic amyotrophic lateral sclerosis. N. Engl. J. Med..

[bib5] Fernandez-Santiago R., Sharma M., Berg D., Illig T., Anneser J., Meyer T., Ludolph A., Gasser T. (2011). No evidence of association of FLJ10986 and ITPR2 with ALS in a large German cohort. Neurobiol. Aging.

[bib6] Fernandez-Santiago R., Garrido A., Infante J., Gonzalez-Aramburu I., Sierra M., Fernandez M., Valldeoriola F., Munoz E., Compta Y., Marti M.J., Rios J., Tolosa E., Ezquerra M., Barcelona L.S.G. (2018). alpha-synuclein (SNCA) but not dynamin 3 (DNM3) influences age at onset of leucine-rich repeat kinase 2 (LRRK2) Parkinson’s disease Spain. Mov. Disord..

[bib7] Foo J.N., Tan L.C., Au W.L., Prakash K.M., Liu J., Tan E.K. (2019). No association of DNM3 with age of onset in Asian Parkinson’s disease. Eur. J. Neurol..

[bib8] Hawrylycz M.J., Lein E.S., Guillozet-Bongaarts A.L., Shen E.H., Ng L., Miller J.A., van de Lagemaat L.N., Smith K.A., Ebbert A., Riley Z.L., Abajian C., Beckmann C.F., Bernard A., Bertagnolli D., Boe A.F., Cartagena P.M., Chakravarty M.M., Chapin M., Chong J., Dalley R.A., David Daly B., Dang C., Datta S., Dee N., Dolbeare T.A., Faber V., Feng D., Fowler D.R., Goldy J., Gregor B.W., Haradon Z., Haynor D.R., Hohmann J.G., Horvath S., Howard R.E., Jeromin A., Jochim J.M., Kinnunen M., Lau C., Lazarz E.T., Lee C., Lemon T.A., Li L., Li Y., Morris J.A., Overly C.C., Parker P.D., Parry S.E., Reding M., Royall J.J., Schulkin J., Sequeira P.A., Slaughterbeck C.R., Smith S.C., Sodt A.J., Sunkin S.M., Swanson B.E., Vawter M.P., Williams D., Wohnoutka P., Zielke H.R., Geschwind D.H., Hof P.R., Smith S.M., Koch C., Grant S.G.N., Jones A.R. (2012). An anatomically comprehensive atlas of the adult human brain transcriptome. Nature.

[bib9] Healy D.G., Falchi M., O'Sullivan S.S., Bonifati V., Durr A., Bressman S., Brice A., Aasly J., Zabetian C.P., Goldwurm S., Ferreira J.J., Tolosa E., Kay D.M., Klein C., Williams D.R., Marras C., Lang A.E., Wszolek Z.K., Berciano J., Schapira A.H., Lynch T., Bhatia K.P., Gasser T., Lees A.J., Wood N.W., International L.C. (2008). Phenotype, genotype, and worldwide genetic penetrance of LRRK2-associated Parkinson’s disease: a case-control study. Lancet Neurol..

[bib10] Hentati F., Trinh J., Thompson C., Nosova E., Farrer M.J., Aasly J.O. (2014). LRRK2 parkinsonism in Tunisia and Norway: a comparative analysis of disease penetrance. Neurology.

[bib11] Kossmeier M. (2019). Visualising meta-analytic data with R package metaviz. https://cran.r-project.org/web/packages/metaviz/vignettes/metaviz.html.

[bib12] Lee A.J., Wang Y., Alcalay R.N., Mejia-Santana H., Saunders-Pullman R., Bressman S., Corvol J.C., Brice A., Lesage S., Mangone G., Tolosa E., Pont-Sunyer C., Vilas D., Schule B., Kausar F., Foroud T., Berg D., Brockmann K., Goldwurm S., Siri C., Asselta R., Ruiz-Martinez J., Mondragon E., Marras C., Ghate T., Giladi N., Mirelman A., Marder K., Michael J.F. (2017). Penetrance estimate of LRRK2 p.G2019S mutation in individuals of non-Ashkenazi Jewish ancestry. Mov. Disord..

[bib13] Malek N., Swallow D.M., Grosset K.A., Lawton M.A., Marrinan S.L., Lehn A.C., Bresner C., Bajaj N., Barker R.A., Ben-Shlomo Y., Burn D.J., Foltynie T., Hardy J., Morris H.R., Williams N.M., Wood N., Grosset D.G., ProBaNd Consortium (2015). Tracking Parkinson’s: study design and baseline patient data. J. Parkinsons Dis..

[bib14] Mata I.F., Checkoway H., Hutter C.M., Samii A., Roberts J.W., Kim H.M., Agarwal P., Alvarez V., Ribacoba R., Pastor P., Lorenzo-Betancor O., Infante J., Sierra M., Gomez-Garre P., Mir P., Ritz B., Rhodes S.L., Colcher A., Van Deerlin V., Chung K.A., Quinn J.F., Yearout D., Martinez E., Farin F.M., Wan J.Y., Edwards K.L., Zabetian C.P. (2012). Common variation in the LRRK2 gene is a risk factor for Parkinson’s disease. Mov. Disord..

[bib15] Nalls M.A., Blauwendraat C., Vallerga C.L., Heilbron K., Bandres-Ciga S., Chang D., Tan M., Kia D.A., Noyce A.J., Xue A., Bras J., Young E., von Coelln R., Simon-Sanchez J., Schulte C., Sharma M., Krohn L., Pihlstrom L., Siitonen A., Iwaki H., Leonard H., Faghri F., Gibbs J.R., Hernandez D.G., Scholz S.W., Botia J.A., Martinez M., Corvol J.C., Lesage S., Jankovic J., Shulman L.M., Sutherland M., Tienari P., Majamaa K., Toft M., Andreassen O.A., Bangale T., Brice A., Yang J., Gan-Or Z., Gasser T., Heutink P., Shulman J.M., Wood N.W., Hinds D.A., Hardy J.A., Morris H.R., Gratten J., Visscher P.M., Graham R.R., Singleton A.B., 23andMe Research., System Genomics of Parkinson's Disease Consortium, International Parkinson's Disease Genomics Consortium (2019). Identification of novel risk loci, causal insights, and heritable risk for Parkinson's disease: a meta-analysis of genome-wide association studies. Lancet Neurol..

[bib16] Ramasamy A., Trabzuni D., Guelfi S., Varghese V., Smith C., Walker R., De T., Coin L., de Silva R., Cookson M.R., Singleton A.B., Hardy J., Ryten M., Weale M.E., Consortium, U.K.B.E., North American Brain Expression Consortium (2014). Genetic variability in the regulation of gene expression in ten regions of the human brain. Nat. Neurosci..

[bib17] Ross O.A., Soto-Ortolaza A.I., Heckman M.G., Aasly J.O., Abahuni N., Annesi G., Bacon J.A., Bardien S., Bozi M., Brice A., Brighina L., Van Broeckhoven C., Carr J., Chartier-Harlin M.C., Dardiotis E., Dickson D.W., Diehl N.N., Elbaz A., Ferrarese C., Ferraris A., Fiske B., Gibson J.M., Gibson R., Hadjigeorgiou G.M., Hattori N., Ioannidis J.P., Jasinska-Myga B., Jeon B.S., Kim Y.J., Klein C., Kruger R., Kyratzi E., Lesage S., Lin C.H., Lynch T., Maraganore D.M., Mellick G.D., Mutez E., Nilsson C., Opala G., Park S.S., Puschmann A., Quattrone A., Sharma M., Silburn P.A., Sohn Y.H., Stefanis L., Tadic V., Theuns J., Tomiyama H., Uitti R.J., Valente E.M., van de Loo S., Vassilatis D.K., Vilarino-Guell C., White L.R., Wirdefeldt K., Wszolek Z.K., Wu R.M., Farrer M.J., Genetic Epidemiology of Parkinson's Disease (2011). Association of LRRK2 exonic variants with susceptibility to Parkinson's disease: a case-control study. Lancet Neurol..

[bib18] Seber G.A.F., Wild C.J. (1989). Nonlinear regression.

[bib19] Therneau T. (2015). A package for survival analysis in S. http://CRAN.R-project.org/package=survival.

[bib20] Trinh J., Gustavsson E.K., Vilarino-Guell C., Bortnick S., Latourelle J., McKenzie M.B., Tu C.S., Nosova E., Khinda J., Milnerwood A., Lesage S., Brice A., Tazir M., Aasly J.O., Parkkinen L., Haytural H., Foroud T., Myers R.H., Sassi S.B., Hentati E., Nabli F., Farhat E., Amouri R., Hentati F., Farrer M.J. (2016). DNM3 and genetic modifiers of age of onset in LRRK2 Gly2019Ser parkinsonism: a genome-wide linkage and association study. Lancet Neurol..

[bib21] Viechtbauer W. (2010). Conducting meta-analyses in R with the metafor package.

