# Supplementary figures and images for: Analysis of DNM3 and VAMP4 as genetic modifiers of LRRK2 Parkinson’s disease
Source: Neurobiol Aging. 2021 Jan;97:148.e17–24. doi: 10.1016/j.neurobiolaging.2020.07.002 (PMC7762821; doi:10.1016/j.neurobiolaging.2020.07.002)

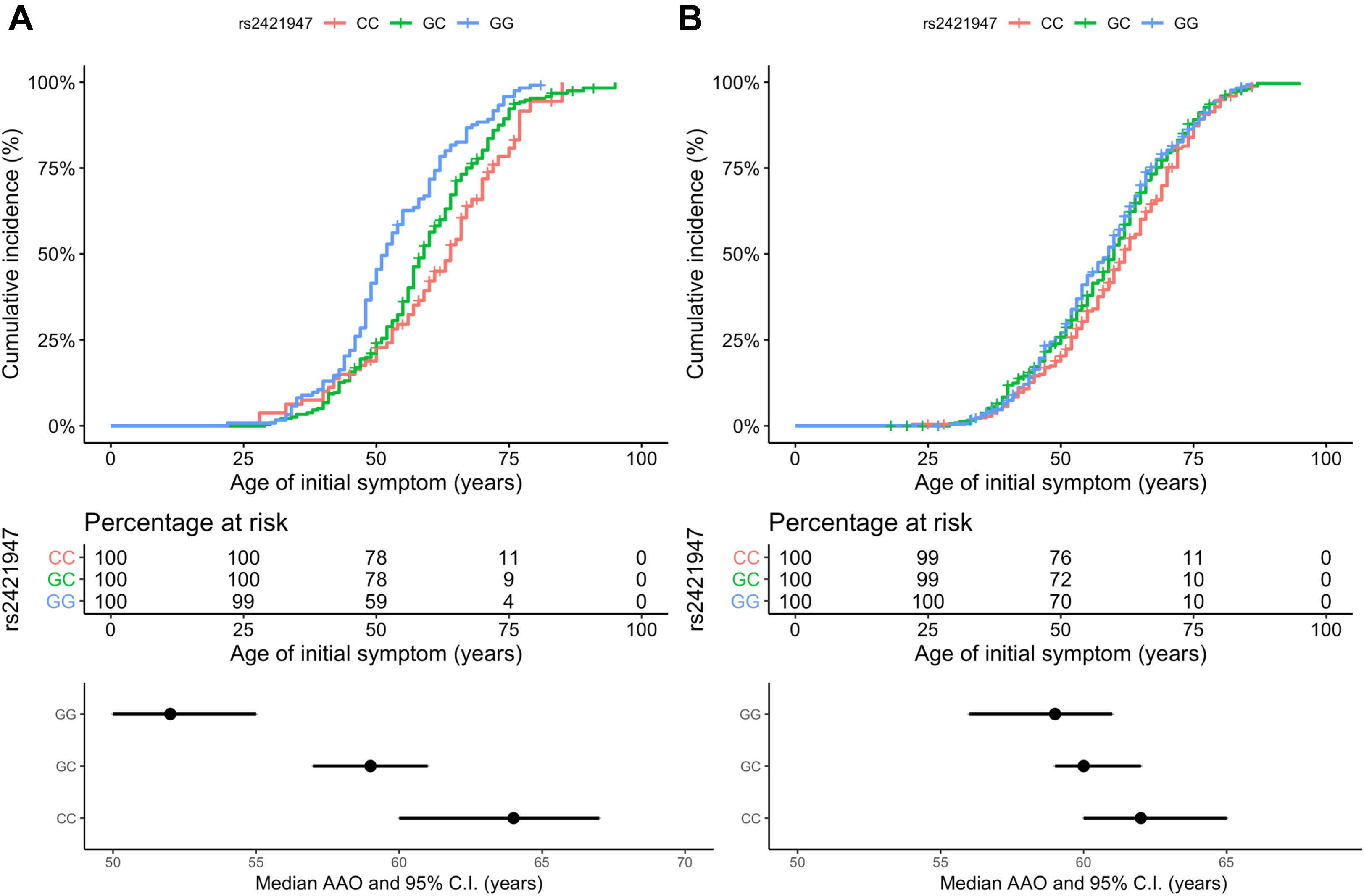

Supplement: Figure E2 — 1 [file figs1.jpg]

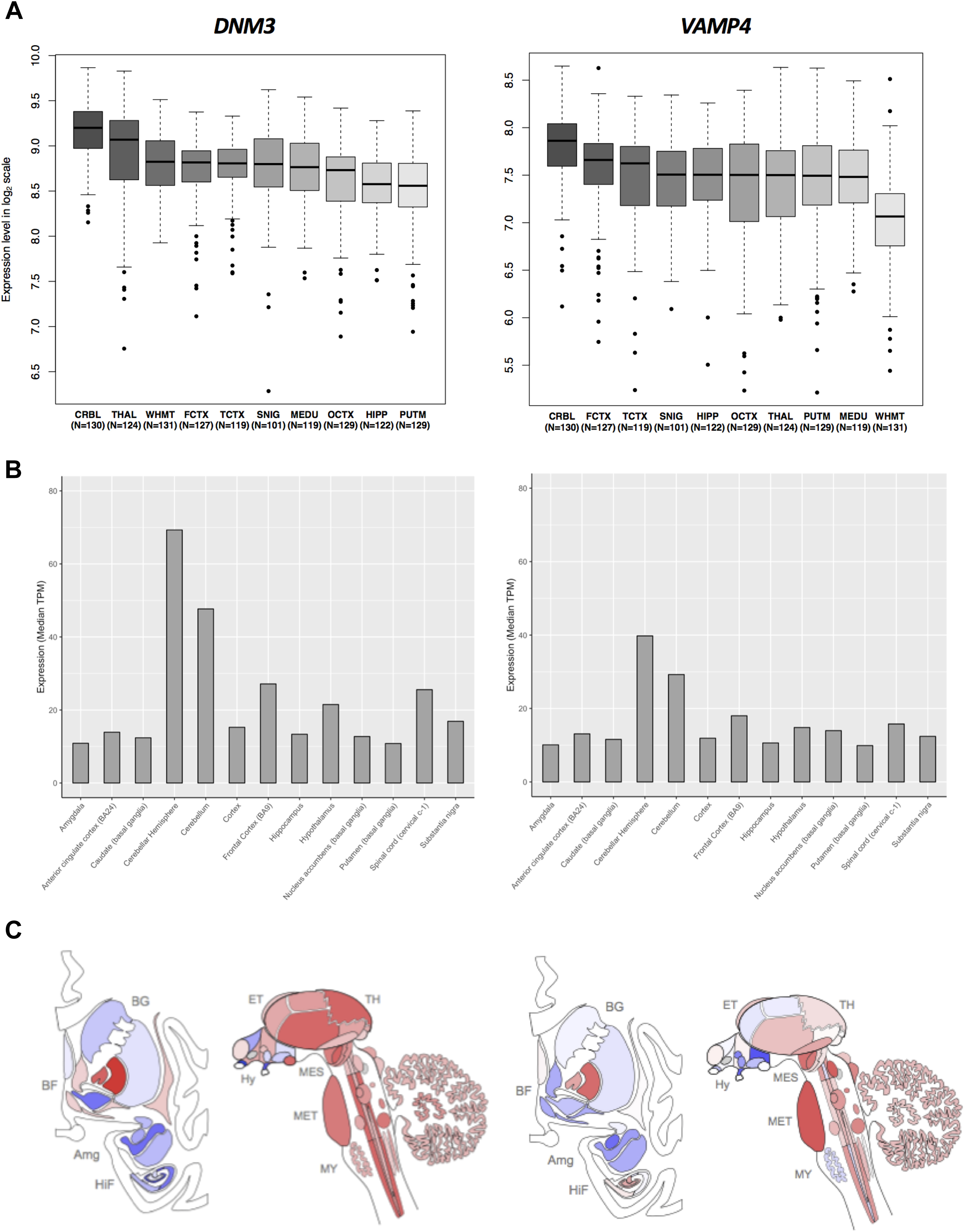

Supplement: Figure E3 — 2 [file figs2.jpg]

...Our

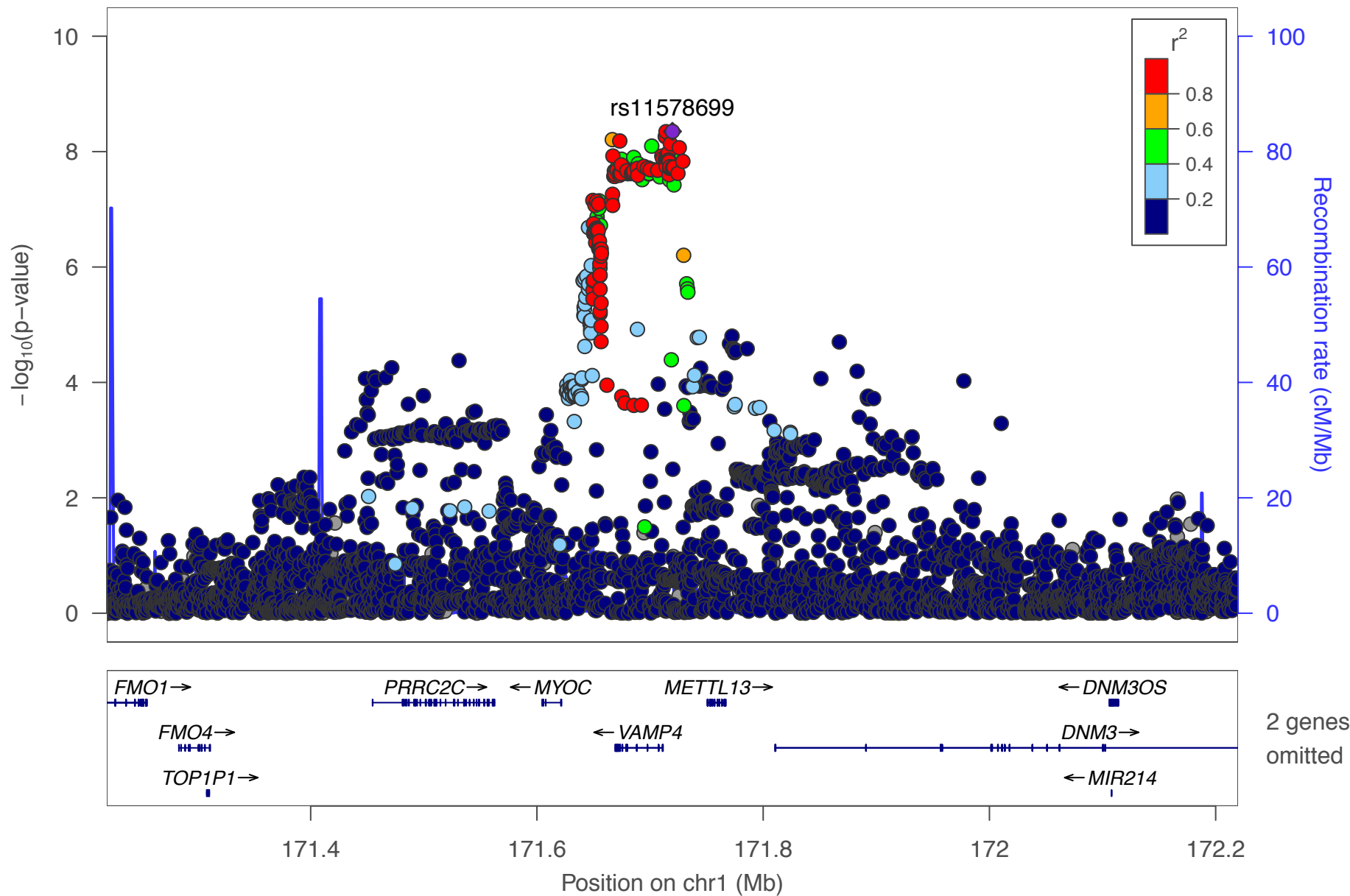

Supplement: Figure E1 [file mmc4.pdf]
