# Supplementary material for: Analysis of DNM3 and VAMP4 as genetic modifiers of LRRK2 Parkinson’s disease
Source: Neurobiol Aging. 2021 Jan;97:148.e17–24. doi: 10.1016/j.neurobiolaging.2020.07.002 (PMC7762821; doi:10.1016/j.neurobiolaging.2020.07.002)
Supplement: Appendix 1 [file mmc1.docx]

**Appendix 1: Authors**

| Name | Location | Role | Contribution |
| --- | --- | --- | --- |
| Emmeline Brown | Department of Movement and Clinical Neurosciences, UCL, London, UK | Author | Software, Formal analysis, Writing - Original Draft, Writing - Review & Editing, Visualization, |
| Cornelis Blauwendraat | Laboratory of Neurogenetics, National Institute on Aging, National Institutes of Health, Bethesda, MD | Author | Methodology, Data Curation, Writing - Review & Editing |
| Joanne Trinh | Institute of Neurogenetics, University of Luebeck, Lübeck, Germany | Author | Data Curation, Writing - Review & Editing |
| Mie Rizig | Department of Movement and Clinical Neurosciences, UCL, London, UK | Author | Resources, Project administration, Writing - Review & Editing |
| Mike Nalls | Laboratory of Neurogenetics, National Institute on Aging, National Institutes of Health, Bethesda, MD | Author | Data Curation, Writing - Review & Editing |
| Etienne Leveille | Department of Human Genetics, McGill University, Montreal, Quebec, Canada | Author | Investigation, Writing - Review & Editing |
| Joanne Ruskey | Department of Human Genetics, McGill University, Montreal, Quebec, Canada | Author | Investigation, Writing - Review & Editing |
| Hallgeir Jonvik | Department of Movement and Clinical Neurosciences, UCL, London, UK | Author | Resources, Writing - Review & Editing, Project administration |
| Manuela Tan | Department of Movement and Clinical Neurosciences, UCL, London, UK | Author | Investigation, Writing - Review & Editing |
| Sara Bandres-Ciga | Laboratory of Neurogenetics, National Institute on Aging, National Institutes of Health, Bethesda, MD, USA | Author | Investigation, Writing - Review & Editing |
| Sharon Hassin-Baer | Sackler Faculty of Medicine, Tel Aviv University, Tel Aviv, Israel | Author | Resources, Funding acquisition, Writing - Review & Editing |
| Kathrin Brockmann | Hertie Institute for Clinical Brain Research and German Center for Neurodegenerative Diseases, University Clinic Tuebingen, Tuebingen, Germany | Author | Resources, Funding acquisition, Writing - Review & Editing |
| Jon Infante | Hospital Universitario Marques de Valdecilla, Spain | Author | Resources, Funding acquisition, Writing - Review & Editing |
| Eduardo Tolosa | Hospital Clinic de Barcelona, Spain | Author | Resources, Funding acquisition, Writing - Review & Editing |
| Mario Ezquerra | Hospital Clinic de Barcelona, Spain | Author | Resources, Funding acquisition, Writing - Review & Editing |
| Sawssan Benromdhan | Research Unit in Neurogenetics, Clinical Investigation Center CIC at the CHU Habib Bourguiba, Sfax, Tunisia | Author | Resources, Funding acquisition, Writing - Review & Editing |
| Mustapha Benmahdjoub | Frantz Fanon hospital, CHU Blida, Algeria | Author | Resources, Funding acquisition, Writing - Review & Editing |
| Chokri Mhiri | Research Unit in Neurogenetics, Clinical Investigation Center CIC at the CHU Habib Bourguiba, Sfax, Tunisia | Author | Resources, Funding acquisition, Writing - Review & Editing |
| Mohammed Arezki | Frantz Fanon hospital, CHU Blida, Algeria | Author | Resources, Funding acquisition, Writing - Review & Editing |
| John Hardy | Department of Movement and Clinical Neurosciences, UCL, London, UK | Author | Funding acquisition, Writing - Review & Editing |
| Andrew Singleton | Laboratory of Neurogenetics, National Institute on Aging, National Institutes of Health, Bethesda, MD, USA | Author | Resources, Funding acquisition, Writing - Review & Editing |
| Roy Alcalay | Department of Neurology, College of Physicians and Surgeons, Columbia University, New York, NY, USA | Author | Resources, Funding acquisition, Writing - Review & Editing |
| Thomas Gasser | Department for Neurodegenerative Diseases, Hertie Institute for Clinical Brain Research, University of Tübingen, Tübingen, Germany | Author | Resources, Funding acquisition, Writing - Review & Editing |
| Donald Grosset | Department of Neurology, Institute of Neurological Sciences, Queen Elizabeth University Hospital, Glasgow, United Kingdom | Author | Resources, Funding acquisition, Writing - Review & Editing |
| Nigel Williams | Division of Psychological Medicine & Clinical Neuroscience, School of Medicine, Cardiff University, Cardiff, UK | Author | Resources, Funding acquisition, Writing - Review & Editing |
| Alan Pittman | Department of Clinical Genetics, St George’s University of London, London SW17 0RE | Author | Methodology, Supervision, Writing - Review & Editing |
| Ziv Gan-Or | Department of Human Genetics, McGill University, Montreal, Quebec, Canada | Author | Resources, Funding acquisition, Writing - Review & Editing |
| Ruben Fernandez-Santiago | Laboratory of Neurodegenerative Disorders, Department of Neurology, Hospital Clínic of Barcelona Institut d'Investigacions Biomèdiques August Pi i Sunyer (IDIBAPS) University of Barcelona (UB), Barcelona, Spain | Author | Resources, Funding acquisition, Writing - Review & Editing |
| Alexis Brice | Research Unit UMR_1127 at Sorbonne Université, Institutet du Cerveau et de la Moëlle épinière (ICM), Paris, France | Author | Resources, Funding acquisition, Writing - Review & Editing |
| Suzanne Lesage | Research Unit UMR_1127 at Sorbonne Université, Institutet du Cerveau et de la Moëlle épinière (ICM), Paris, France | Author | Resources, Funding acquisition, Writing - Review & Editing |
| Matthew Farrer | Department of Medical Genetics, University of British Columbia, Vancouver, Canada | Author | Conceptualization, Resources, Funding acquisition,  Writing - Review & Editing |
| Nicholas Wood | Department of Movement and Clinical Neurosciences, UCL, London, UK | Author | Conceptualization, Resources, Supervision, Project administration, Funding acquisition,  Writing - Review & Editing |
| Huw Morris | Department of Movement and Clinical Neurosciences, UCL, London, UK | Author | Conceptualization, Methodology, Resources, Writing - Review & Editing, Supervision, Project administration, Funding acquisition |
