# Supplementary material for: Analysis of DNM3 and VAMP4 as genetic modifiers of LRRK2 Parkinson’s disease
Source: Neurobiol Aging. 2021 Jan;97:148.e17–24. doi: 10.1016/j.neurobiolaging.2020.07.002 (PMC7762821; doi:10.1016/j.neurobiolaging.2020.07.002)
Supplement: Appendix 2 [file mmc2.docx]

**Appendix 2: Co-investigators**

| Name | Location | Role | Contribution |
| --- | --- | --- | --- |
| J Raphael Gibbs | National Institute on Aging, USA | Co-investigator | IPDGC member |
| Dena G Hernandez | National Institute on Aging, USA | Co-investigator | IPDGC member |
| Xylena Reed | National Institute on Aging, USA | Co-investigator | IPDGC member |
| Mark R Cookson | National Institute on Aging, USA | Co-investigator | IPDGC member |
| Faraz Faghri | National Institute on Aging, USA | Co-investigator | IPDGC member |
| Hampton L. Leonard | National Institute on Aging, USA | Co-investigator | IPDGC member |
| Hirotaka Iwaki | National Institute on Aging, USA | Co-investigator | IPDGC member |
| Joshua M. Shulman | Baylor College of Medicine, USA | Co-investigator | IPDGC member |
| Laurie Robak | Baylor College of Medicine, USA | Co-investigator | IPDGC member |
| Codrin Lungu | National Institute of Health, USA | Co-investigator | IPDGC member |
| David W. Craig | University of Southern California, USA | Co-investigator | IPDGC member |
| Kendall Van Keuren-Jensen | TGen, USA | Co-investigator | IPDGC member |
| Sonja Scholz | National Institute of Neurological Disorders and Stroke, USA | Co-investigator | IPDGC member |
| Steven Finkbeiner | University of California, USA | Co-investigator | IPDGC member |
| Steven Lubbe | Northwestern University Feinberg School of Medicine, USA | Co-investigator | IPDGC member |
| Niccolo E. Mencacci | Northwestern University Feinberg School of Medicine, USA | Co-investigator | IPDGC member |
| Helene Plun-Favreau | UCL Institute of Neurology, UK | Co-investigator | IPDGC member |
| Jose M Bras | UCL Institute of Neurology, UK | Co-investigator | IPDGC member |
| Kin Y Mok | UCL Institute of Neurology, UK | Co-investigator | IPDGC member |
| Lea R’Bibo | UCL Institute of Neurology, UK | Co-investigator | IPDGC member |
| Mina Ryten | UCL Institute of Neurology, UK | Co-investigator | IPDGC member |
| Rita Guerreiro | UCL Institute of Neurology, UK | Co-investigator | IPDGC member |
| Alastair J Noyce | UCL Institute of Neurology, UK | Co-investigator | IPDGC member |
| Sebastian Guelfi | UCL Institute of Neurology, UK | Co-investigator | IPDGC member |
| Rauan Kaiyrzhanov | UCL Institute of Neurology, UK | Co-investigator | IPDGC member |
| Viorica Chelban | UCL Institute of Neurology, UK | Co-investigator | IPDGC member |
| Sonia Garcia Ruiz | UCL Institute of Neurology, UK | Co-investigator | IPDGC member |
| Henry Houlden | UCL Institute of Neurology, UK | Co-investigator | IPDGC member |
| Peter Holmans | MRC Centre for Neuropsychiatric Genetics & Genomics, Cardiff, UK | Co-investigator | IPDGC member |
| John Quinn | University of Liverpool, UK | Co-investigator | IPDGC member |
| Kimberley Billingsley | University of Liverpool, UK | Co-investigator | IPDGC member |
| Ben Middlehurst | University of Liverpool, UK | Co-investigator | IPDGC member |
| Valentina Escott-Price | Cardiff University School of Medicine, UK | Co-investigator | IPDGC member |
| Nigel Williams | Cardiff University School of Medicine, UK | Co-investigator | IPDGC member |
| Raquel Real | UCL Institute of Neurology, UK | Co-investigator | IPDGC member |
| Demis Kia | UCL Institute of Neurology, UK | Co-investigator | IPDGC member |
| Thomas Foltynie | UCL Institute of Neurology, UK | Co-investigator | IPDGC member |
| Sebastian Schreglmann | UCL Institute of Neurology, UK | Co-investigator | IPDGC member |
| Ruth Lovering | UCL, UK | Co-investigator | IPDGC member |
| Patrick Lewis | University of Reading, UK | Co-investigator | IPDGC member |
| Claudia Manzoni​ | University of Reading, UK | Co-investigator | IPDGC member |
| Peter Heutink | University of Tübingen, Germany | Co-investigator | IPDGC member |
| Javier Simón-Sánchez | University of Tübingen, Germany | Co-investigator | IPDGC member |
| Patrizia Rizzu | University of Tübingen, Germany | Co-investigator | IPDGC member |
| Manu Sharma | University of Tübingen, Germany | Co-investigator | IPDGC member |
| Claudia Schulte | University of Tübingen, Germany | Co-investigator | IPDGC member |
| Fabrice Danjou | Sorbonne Universités, UK | Co-investigator | IPDGC member |
| Jean-Christophe Corvol | Sorbonne Universités, UK | Co-investigator | IPDGC member |
| Maria Martinez | Paul Sabatier University, France | Co-investigator | IPDGC member |
| Guy A. Rouleau | McGill University, Canada | Co-investigator | IPDGC member |
| Lynne Krohn | McGill University, Canada | Co-investigator | IPDGC member |
| Pille Taba | University of Tartu, Estonia | Co-investigator | IPDGC member |
| Jacobus J van Hilten | Leiden University Medical Center, Netherlands | Co-investigator | IPDGC member |
| Johan Marinus | Leiden University Medical Center, Netherlands | Co-investigator | IPDGC member |
| Lasse Pihlstrom | Oslo University Hospital, Norway | Co-investigator | IPDGC member |
| Alexander Zimprich | Medizinische Universität, Austria | Co-investigator | IPDGC member |
| Juan A. Botía | Universidad de Murcia, Spain | Co-investigator | IPDGC member |
| Sulev Koks | Perron Institute for Neurological and Translational Science, Australia | Co-investigator | IPDGC member |
