# Supplementary material for: Analysis of DNM3 and VAMP4 as genetic modifiers of LRRK2 Parkinson’s disease
Source: Neurobiol Aging. 2021 Jan;97:148.e17–24. doi: 10.1016/j.neurobiolaging.2020.07.002 (PMC7762821; doi:10.1016/j.neurobiolaging.2020.07.002)
Supplement: Supplementary methods [file mmc3.docx]

**Supplementary methods**

Blood samples from UCL studies and the *Tracking Parkinson’s* study were collected at study entry. DNA was extracted from an ethylene diamine tetraacetic acid (EDTA) sample. All patients from UCL were assessed for *LRRK2* G2019S mutation status and *DNM3* rs2421947 using the ‘Kompetitive’ allele-specific polymerase chain reaction (KASP) assay (LGC Genomic Solution. Data was validated using the Truseq Targeted Neurodegeneration Illumina panel or the Illumina NeuroChip array, a custom array with backbone Infinium HumanCore-24 v1.0. 100% of UCL samples were successfully genotyped or imputed with imputation quality indicated in table e-1. Samples genotyped at McGill University were sequenced using a Taqman assay (Assay ID - C__15804853_10) with 100% call rate. DNA samples coordinated by Sorbonne University were collected both as saliva using Oragene and extraction with the PrepIT-L2P (DNA Genotek) purification kit and DNA from blood samples extracted with the phenol-chloroform protocol. The integrity of all samples was checked by agarose gel electrophoresis and DNA at 200ng/ µl was stored at -20 °C. *LRRK2* G2019S was screened by TaqMan qPCR assays in all patients and validated by Sanger sequencing. All samples were successfully genotyped. Study size was determined using all available data from the IPDGC and collaborative centres willing to participate. For the Sorbonne University study recruitment was 8 years in duration from 2006 to 2013 and patients continue to be followed up.

Age-at-onset was self-defined by patients across studies including those previously reported by Fernandez et al (2018) and Trinh et al (2016). Age-at-onset data was defined based on patient report of initial manifestation of Parkinsonian motor symptoms (tremor, bradykinesia, rigidity, or gait impairment). Overall 10 cases were found to have the same age-at-exam as age-at-onset, indicating correct ascertainment of an earlier disease onset date for cases.

For the meta-analyses data was divided into the same ethnicity subgroups (Ashkenazi Jewish, North African, and European using PCA and self-report where this was missing), and study centre specific groups. Where there were <60 datapoints from a particular study centre for *DNM3* rs2421947, data was pooled, and study centre of origin was used as a covariate. Cox proportional hazards model analyses and linear regressions for p.G2019S carriers were carried out on GG versus CC and CG genotypes, as exploratory data analysis indicated a dominant protective effect of the minor C allele. For the *VAMP4* rs11578699 variant these survival methods were carried out on CC versus TC and TT due to the smaller number of TT genotypes available. Family relatedness, sex, and principal components of ancestry were used as covariates, where available, in each analysis.

A random-effects model was used in meta-analyses. Natural logs of hazard ratios for GG versus CC and CG genotypes were taken for *DNM3* rs2421947, and for CC versus TC and TT for *VAMP4* rs11578699. The log of standard errors was calculated using outputted hazard ratio confidence intervals, using the Taylor expansion with leading term (also known as the delta method) (Seber, 1989).

RSQ and D’ were calculated in PLINK 1.9 (Chang et al., 2015). The allele based Fisher’s Exact test, ANOVA, Student’s t tests, and linear regressions (PD onset age regressed on *DNM3* rs2421947 genotype, and separately *VAMP4* rs11578699 genotype) were performed in base R RStudio. Cox proportional hazards survival analysis and Kaplan Meier curves were carried out using the Terry Therneau “Survival” package (Therneau, 2015) in RStudio. Time-to-event in survival analyses was the self-reported age at which a participant developed motor symptoms of PD. For p.G2019S carriers without PD time-to-event was right-censored from sampling age. Meta-analysis was carried out in metafor (Viechtbauer, 2010) and metaviz R packages (Kossmeier, 2019).

We supplemented the analysis of *LRRK2* p.G2019S by analysing time-to-event (by *DNM3* rs2421947 and *VAMP4* rs11578699) genotypes in PD cases carrying the *LRRK2* risk variant rs10878226 (Mata et al., 2012). We identified 4882 PD cases heterozygous or homozygous for the minor risk allele from the 21,242 PD cases in IPDGC datasets. Linear regression of AAO, Kaplan Meier and Cox proportional hazards analyses were then performed on *VAMP4* rs11578699 genotypes against age at onset. These methods were carried out on TT versus TC and CC genotypes as exploratory data analysis indicated an effect of the homozygous T allele on AAO. Covariates of sex, ethnicity (PC1-PC10) and dataset were used. We carried out the same analysis on 14970 PD cases not carrying the *LRRK2* rs10878226 variant.

**Gene Expression**

The BRAINEACv2 database hosts data on brain tissues (frontal cortex, temporal cortex, parietal cortex, occipital cortex, hippocampus, thalamus, putamen, substantia nigra, medulla, cerebellum, and white matter) from 134 healthy controls. The GTEx database consists of 8555 samples from 53 tissues (including 13 brain regions) from 544 donors. The Allen Human Brain Atlas database microarray data is from 8 neuropathologically normal individuals of varying ethnicity, and covers ~150 brain regions.

**Supplementary results**

**Cox proportional hazard analysis**

*DNM3* rs2421947 and V*AMP4* rs11578699 genotypes were in Hardy-Weinberg equilibrium in controls.

There was no LD between *DNM3* rs2421947 and *VAMP4* rs11578699, as assessed through RSQ and D’, within or across population samples, indicating that these are independent variants (table e-2).

We investigated the possibility that ethnic variation in allele frequency might explain a variable effect of *DNM3* on affected *LRRK2* penetrance. Aside from the Norwegian cohort *DNM3* rs2421947 MAF (C) of 0.69 (vs. other Europeans p=0.00012), all *DNM3* rs2421947 MAF in the study were between 0.43-0.51 (table 1). There was no difference between Ashkenazi Jews and European allele frequencies (p=0.94); or between Ashkenazi Jews and North Africans (p=0.28); nor between Europeans and North Africans (p=0.54) at this locus. Publicly available allele frequencies (http://gnomad.broadinstitute.org) were similar (table 1). *VAMP4* rs11578699 MAF were between 0.15-0.30 in different populations.

We assessed possible association between these variants and p.G2019S genotype using the allele based Fisher’s exact test. There was no association between *DNM3* rs2421947 and p.G2019S genotype in all PD cases (p=0.42).There was no significant difference in *DNM3* allele frequencies between patients with PD and asymptomatic heterozygotes (asymptomatic p.G2019S heterozygotes were of Ashkenazi Jewish, North African, and European non-Jewish ethnicity), as assessed through Fisher’s Exact test (p=0.056). When the previous data was meta-analyzed with the current data there was a significant association between *DNM3* rs2421947 and PD affected status in p.G2019S heterozygotes (p=3.1x10^-5^) by the Fisher’s Exact test, although there were differences between populations (Table 1). Mean sampling age for p.G2019S heterozygotes without PD was 56 years across all ethnicities; 63 years in AJ subjects; and 56 years for Europeans.

In the most recent PD GWAS there was no genome-wide significant association between *DNM3* rs2421947 and PD by logistic regression, (p=0.0051). There was no association between *VAMP4* rs11578699 and *LRRK2* p.G2019S status in European PD (p=0.60) and no significant difference in *VAMP4* rs11578699 MAF between *LRRK2* p.G2019S patients and asymptomatic carriers by the Fisher’s Exact test (p=0.64).

We then assessed the relationship between AAO and *DNM3* rs2421947 and *VAMP4* rs11578699 (table 2) using t-tests and ANOVA. There was no effect of *DNM3* rs2421947 on AAO as assessed through ANOVA (p=0.55, F=0.59, df=2, n=708). When meta-analysed with data from previous cohorts there was a nominal effect of *DNM3* on AAO (2 years difference between G and C genotypes), (p=0.021, F=3.88, df=2, n=1304).

Sub-analyses confirmed that genetic heterogeneity between Northern and Southern Europeans has not contributed to the non-replication in these data for *LRRK2* p.G2019S heterozygotes. *LRRK2* p.G2019S heterozygote Southern Europeans (defined by principal components of ancestry and self-report), *DNM3* rs2421947 was not associated with PD risk for GG versus GC and CC carriers (beta = -0.70, se = 1.58,p = 0.67; hazard ratio [HR] 0.96, 95% CI 0.73-1.26, p = 0.75, n = 387). *DNM3* rs2421947 was also not associated with PD risk for North Europeans GG versus GC and CC carriers (beta = -2.44, se = 2.17, p = 0.31; hazard ratio [HR] 1.20 95% 0.93-1.62, p = 0.76, n = 458). Heterogeneity in the European cohort of idiopathic PD cases has been assessed in the genome-wide association study (Nalls et al., 2019).

*DNM3* did not influence time to the development of disease in idiopathic PD by the Cox proportional hazards model (hazard ratio [HR] 0.98, 95% CI 0.89-1.07, p = 0·60, n=1956) for GG versus CC and CG carriers. *DNM3* rs2421947 did not significantly affect the age associated hazards of developing p.G2019S parkinsonism through random effects meta-analysis in our previously unpublished data (hazard ratio [HR] 1.09, 95% CI 0.95-1.25, p = 0.20, n=724), as shown in Figure 2 . I^2^ heterogeneity was <0.01% (p = 0.42). When our new data was pooled with the two previous studies and meta-analysed, there was a nominally significant effect of *DNM3* rs2421947 on AAO in *LRRK2* p.G2019S parkinsonism ([HR] 1.14, 95% CI, 1.02-1.27, p = 0.025, n = 1478).  I^2^ total heterogeneity was 22.8%, p=0.37.

*VAMP4* rs11578699 was nominally associated with disease AAO in idiopathic PD cases carrying the *LRRK2* risk variant rs10878226 (TT versus CC and TC (hazard ratio [HR] 0.86, 95% CI 0.76-0.98, p=0.023), and not associated with AAO in idiopathic PD cases not carrying the *LRRK2* risk variant rs10878226 (hazard ratio [HR] 1.02, 95% CI 0.94-1.10, p=0.47).

We reviewed *VAMP4* and *DNM3* human brain gene expression and co-expression data from the BRAINEACv2^23^ (Ramasamy et al., 2014), GTEx, and Allen Atlas databases (Hawrylycz et al., 2012) *(*figure e-3)*. VAMP4* rs11578699 is an eQTL for *VAMP4* expression: NES: 0.29, p = 3.5x10^-9^ (cerebellum, GTEx database); NES: 0.32, p = 6.5x10^-7^ (cerebellar hemisphere, GTEx database). *DNM3* rs2421947 did not appear to be an eQTL for *DNM3* expression in the brain in the GTex database, though *VAMP4* and *DNM3* gene expression in brain were high (figure e-3).

**Supplementary figure and table legends**

**Table e-1. Imputation quality per subgroup**

**Table e-2. Population specific D’ and RSQ values between *DNM3* rs2421947 and *VAMP4* rs11578699**

**Table e-3. Population series mean and standard deviation age at onset in years of p.G2019S PD cases for rs2421947 genotypes; One-way ANOVA between rs2421947 genotypes; Student’s t test between rs2421947 genotypes**

**Figure e-1 figure legend. Regional association plot of PD cases versus control from the largest PD GWAS, identifying rs11578699 as the lead SNP**

**figure e-2 figure legend. Kaplan Meier plots by rs2421947 genotypes- comparison of discovery data and subsequent cohorts.**

(A) Discovery data (n = 440) p.G2019S carriers Kaplan Meier plot and median plot (B) Subsequent cohorts (n = 1038) p.G2019S carriers Kaplan Meier plot and median plot.

**figure e-3 figure legend. Regional variation in the brain of *DNM3* and *VAMP4***

DNM3 and VAMP4 brain expression in: **A.** BRAINEAC database – CRBL = Cerebellum, PUTM = Putamen, HIPP = Hippocampus, TCTX = Temporal cortex, OCTX = Occipital cortex, FCTX = Frontal cortex, THAL = Thalamus, SNIG = Substantia nigra, MEDU = Medulla, WHMT = White matter; **B.** GTEx database; **C.** Allen Atlas database (European subjects) – Amg = Amygdala, BF = Basal Forebrain, BG = Basal Ganglia, CAU = Caudate, ET = Epithalamus, HiF = Hippocampal Formation, Hy = Hypothalamus, MES = Mesencephalon, MET = Metencephalon, MY = Myelencephalon, PUT = Putamen, TH = Thalamus. Image credit: Allen Institute.

**Table e-1.**

| **RSQ imputation score** |  |
| --- | --- |
| **iPD *DNM3* rs2421947** | |
| >0.99 | 100% |
| **p.G2019S carriers *DNM3* rs2421947** | |
| 1.00 | 70% |
| >0.99 | 73% |
| >0.90 | 98% |
| **p.G2019S carriers *VAMP4* rs11578699^1^** |  |
| 1.00 | 79% |
| >0.98 | 86% |
| >0.75 | 100% |
| **iPD *VAMP4* rs11578699** | |
| Previously published PD GWAS data^1^ | 100% |

**Table e-2.**

|  | **Controls** | **PD cases** | **p.G2019S PD cases** |
| --- | --- | --- | --- |
| **D’** | | | |
| **Ashkenazi Jewish** | N/A | N/A | 0.087 (n=29) |
| **African/North African** | 0.089 (n=1322)**^a^** | N/A | 0.061 (n=482) |
| **From Spain/Latino** | 0.30 (n=214)^b^ | N/A | N/A |
| **European non-Finnish** | 0.46 (n=594)^c^ | 0.21 (n=14508) | 0.27 (n=186) |
| **Total** | 0.22 (n=2130) | 0.21 (n=14508) | 0.021 (n=697) |
| **RSQ** | | | |
| **Ashkenazi Jewish** | N/A | N/A | 0.0035 (n=29) |
| **African/North African** | 0.0013 (n=1322)**^a^** | N/A | 0.0022 (n=482) |
| **From Spain/Latino** | 0.037 (n=214)^b^ | N/A | N/A |
| **European non-Finnish** | 0.039 (n=594)^c^ | 0.0086 (n=14508) | 0.017 (n=186) |
| **Total** | 0.0085 (n=2130) | 0.0086 (n=14508) | 0.000098 (n=697) |

^a^All (AFR) Populations: (YRI, LWK, GWD, MSL, ESN, ASW, ACB)

^b^Selected (EUR) population: (IBS)

^c^Selected (EUR) population: (CEU, GBR, TSI)

**Table e-3.**

| **Population series** | **Mean age at onset (sd)** | | | **ANOVA p value** | **t test GG versus CG p value^b^** | **t test CG versus CC p value^b^** | **t test GG versus CC p value^b^** |
| --- | --- | --- | --- | --- | --- | --- | --- |
|  | **GG** | **CG** | **CC** |  |  |  |  |
| **European unspecific (n=239)^a^** | 65.35  (11.15) | 62.19 (12.19) | 62.51 (14.00) | 0.23 | 0.075 | 0.88 | 0.24 |
| **American (n=87)** | 56.30  (7.53) | 61.76 (10.14) | 64.55 (10.98) | 0.16 | 0.061 | 0.44 | 0.058 |
| **French (n=98)** | 52.08  (9.96) | 50.93 (13.48) | 49.94 (10.51) | 0.81 | 0.66 | 0.77 | 0.49 |
| **Norwegian (n=18)** | 44.00  (N/A) | 72.55 (15.12) | 61.67 (20.81) | 0.21 | Insufficient count^c^ | 0.29 | Insufficient count^c^ |
| **Spanish (n=195)** | 58.70  (14.01) | 59.93 (12.59) | 60.24 (12.78) | 0.81 | 0.60 | 0.89 | 0.56 |
| **British (n=36)** | 49.68  (9.54) | 53.78  (11.20) | 60.00  (9.99) | 0.13 | 0.32 | 0.18 | 0.042 |
| **Ashkenazi Jewish (n=146)** | 57.20  (8.96) | 60.11  (12.21) | 58.41 (8.01) | 0.34 | 0.13 | 0.45 | 0.58 |
| **North African (n=480)** | 53.02  (11.89) | 54.16 (11.41) | 55.75 (12.11) | 0.23 | 0.33 | 0.32 | 0.10 |

^a^Samples defined as from a non-specific European background (through PCA or self-identification during clinician interview).

^b^Uncorrected for multiple testing

^c^There are insufficient p.G2019S carriers in these categories for the t test to be calculated.
